# Effects of pregnancy and pregnancy-related conditions on the inner ear: a systematic review

**DOI:** 10.1017/S002221512510354X

**Published:** 2025-12

**Authors:** Sara Timms, Emma Stapleton

**Affiliations:** 1 Department of Otolaryngology, Manchester Royal Infirmary, Manchester, UK; 2 University of Manchester Medical Academic Health Science Centre, Manchester, UK

**Keywords:** balance, cochlea, gestational diabetes, hearing, hearing loss, inner ear, pregnancy, pre-eclampsia, vestibular labyrinth

## Abstract

**Introduction:**

The inner ear is a complex sensory organ with finely balanced physiology; disrupting this may cause hearing changes or vestibular symptoms. Pregnancy involves multiple significant reversible alterations in physiological state. This study reviews literature on the inner ear in pregnancy.

**Methods:**

The review was pre-registered on the PROSPERO database CRD42023446898. Robust searches were conducted by two independent researchers according to the PRISMA 2020 guideline.

**Results:**

A total of 69 studies were filtered into the final analysis. Consistent evidence of subclinical hearing loss in pregnancy was identified, which resolved following childbirth. Auditory processing is affected by pregnancy. Vestibular dysfunction may contribute to pregnancy nausea. Sudden sensorineural hearing loss does not occur more frequently in pregnancy.

**Conclusion:**

This review summarises evidence for reversible and irreversible changes to hearing and vestibular function in pregnancy and pregnancy-related conditions, reviewing aetiological theories and offering insight to audiovestibular physiology and explaining audiovestibular symptoms in the pregnant patient.

## Introduction

The inner ear is a delicate organ, its function reliant on a fine balance of electrolyte and fluid content in the endolymph and peri-lymph compartments. Hearing thresholds fluctuate during the menstrual cycle,^1^ the pattern of which suggests an otoprotective effect of circulating oestrogen,^2^ corroborated by studies comparing hearing in peri-menopausal women taking hormone replacement therapy (HRT) and those not taking HRT.^3^ Vestibular function similarly shows hormonal cycle variability,^4^ and pre-menstrual exacerbation of vertigo in Menière’s disease has also been reported.^5^

In pregnancy, persistent high circulating levels of oestrogen and progesterone lead to more marked physiological changes than those of the menstrual cycle. Cardiac output and circulating volume increase, haemodilution occurs, a prothrombotic state develops and the immune systems adapts to become “tolerant” of the developing fetus. These systemic changes have all been hypothesised to explain the effects of pregnancy on inner ear function.^6^^–^^8^

Sensorineural hearing loss and vestibular loss of function caused by trauma, ototoxicity and ageing are understood to be irreversible, whereas other inner ear conditions, such as sudden sensorineural hearing loss (SSNHL), or the hearing loss of early Menière’s disease, demonstrate a degree of reversibility. The phenomenon of inner ear dysfunction reversibility has had little attention in the literature, and so pregnancy, with its physiological changes that are reversed post-partum, offers a unique insight into this aspect of vestibulocochlear function.

The aim of this review was to evaluate literature reporting the effect of pregnancy and pregnancy syndromes on the function of the inner ear. Initial searching revealed a heterogeneity of publications that were largely observational studies and reviews, and so a systematic review approach was employed.

## Material and methods

The review protocol was pre-registered on the PROSPERO system (CRD42023446898). Two authors independently searched the following databases: PubMed, Embase, CINAHL and the Cochrane Library. The search terms used were (pregnan* OR gestational diabetes OR pre-eclampsia OR preeclampsia) AND (auditory OR hearing OR vestibular OR tinnitus OR misophonia OR phonophobia OR hyperacusis OR decreased sound tolerance). The databases were initially accessed on August 2nd 2023 and were last accessed on 23 January 2025. Abstracts were screened by both authors to exclude duplicates and manuscripts not relevant to the present review. Filtered abstracts then underwent full-text analysis. Further relevant articles were identified through the screening of reference lists from included articles.

The eligibility criteria were broad: any study clearly demonstrating the effect of pregnancy or pregnancy-related conditions on the inner ear was included. Case studies were included, as were pieces of original research and reviews. The exclusion criteria were limited to studies not in the English language and animal studies. There was no specified time frame, all years of publication were considered. This extensive search methodology aimed to find all original research published on the subject. The authors were conscious that randomised controlled trials in pregnancy are rare and that the literature was likely to be circumstantial, descriptive and observational, with case studies a common format. With this understanding, qualitative analysis rather than meta-analysis was anticipated. Analysis was undertaken according to the PRISMA (Preferred Reporting Items for Systematic Reviews and Meta-Analyses) statement.^9^

Risk of bias is high in case studies and studies with small participant numbers, and this was taken into consideration in the qualitative analysis.

## Results and analysis

**Figure 1.**
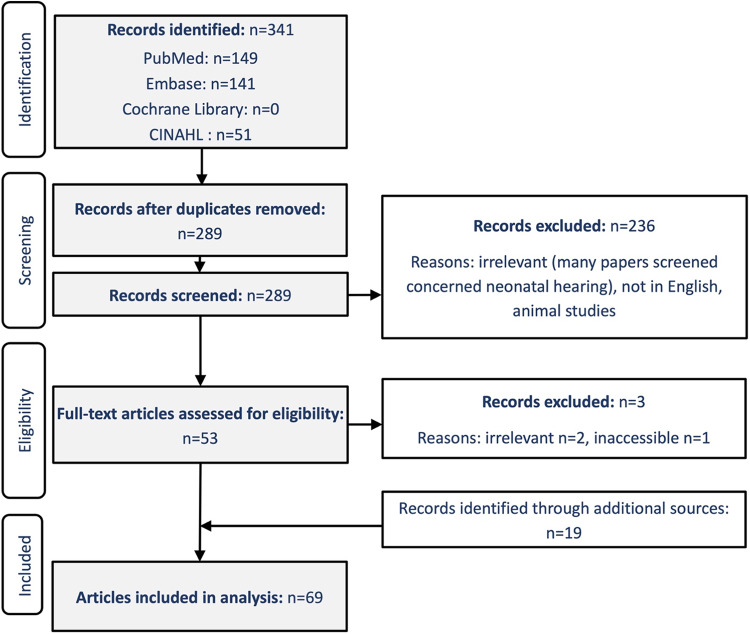
PRISMA flow diagram displaying the systematic search methodology.

Following the pre-registered PROSPERO protocol, initial searching yielded 341 records. The majority were found through PubMed and Embase, with no Cochrane reviews found. Fifty-two of these records were duplicates, and 236 were removed based on exclusion criteria. The search terms yielded many articles about neonatal hearing, as influenced by conditions during pregnancy, and these were excluded as irrelevant from this review. Non-English language studies and animal studies were also excluded. Of the remaining 53 articles, three were excluded following full-text review (one inaccessible, two irrelevant). An additional 19 articles were included through screening references; therefore, in total, 69 articles were included in the review. This process is summarised in the PRISMA diagram (Figure 1).

### Study characteristics

Of the 69 articles reviewed, 37 were research, of which 29 were prospective observational studies and eight were retrospective reviews. There were no interventional randomised controlled trials, but many observational studies included matched controls. Of the remaining 32 articles, nine were case series and 14 were individual case studies. There were seven reviews, one descriptive chapter and one published comment on another study.

Forty-one of the 69 articles were published in the ENT and audiology literature (59 per cent), while 17 appeared in obstetric journals (25 per cent). The remainder were published in journals of general medicine (4), medical sciences (3), neurosurgery (2) and audiovestibular medicine (2).

Qualitative analysis involved reviewing the published work in two broad categories: those that assessed the effects on the inner ear of normal pregnancy (often with non-pregnant controls) and those that considered the effect of pregnancy syndromes on the inner ear (often with healthy pregnant controls).

### Normal pregnancy and the inner ear

The effect of a healthy pregnancy on the inner ear function was the subject of 57 of the included articles. These dealt with a variety of subjects, including hearing physiology, higher auditory processing, vestibular function, hearing disorders, vestibular disorders and cerebellopontine angle lesions. The main inner ear disorder considered in the context of pregnancy was SSNHL, being the subject of one prospective study, seven retrospective studies, five case series and eight other articles.

#### Hearing

A study that performed pure-tone audiometry (PTA) on women in each trimester of pregnancy and post-partum, found statistically significant increases in the lower-frequency hearing thresholds in pregnancy.^10^ These amounted to around 5 decibels poorer hearing in the third trimester compared with peri-partum and non-pregnant controls, and a similar study found even greater hearing differences, again in the low frequencies.^11^ A third study from India found a statistically significant 3dB improvement in average PTA threshold (500, 1000 and 2000Hz) peri-partum when compared with the third trimester.^12^ All three studies found that there was reversibility to this subclinical hearing loss in pregnancy.

A Danish retrospective study reviewed 144 women who had had unilateral stapedectomy for otosclerosis and found that 24 per cent reported that their hearing loss had started or worsened in pregnancy and that a statistically significant hearing deterioration occurred peri-operatively in patients who had a subsequent pregnancy.^13^ Only air conduction levels are reported, but this may suggest a sensorineural effect of pregnancy worsening the symptoms of otosclerosis and not appearing to show reversibility. A case report from Bhutan described a rare cause of hearing loss in pregnancy: typhus.^14^ The authors suggest that changes in immune state in pregnancy may be the reason why hearing loss was permanent in this case.

A small study with 100 subjects that tested distortion-product otoacoustic emissions (DPOAEs) found them to be absent in a much higher proportion of pregnant than non-pregnant subjects (26 per cent compared with 4 per cent; *p* = 0.0038), all of whom had normal PTA findings.^15^

A questionnaire^16^ found a higher incidence of self-reported aural fullness and tinnitus in pregnant women than non-pregnant controls, particularly in those with low blood pressure. This was supported by a study reviewing 84 presentations of pregnant women to ENT: the most common symptom was the sensation of ear blockage, and 21 per cent of these patients had a clinical hearing loss.^17^

One study^18^ reviewing the hearing of five cochlear implant users during pregnancy found that one had to increase the T-level (the weakest electrical signal that elicits an auditory response) during pregnancy and breastfeeding, indicating that this patient’s hearing deteriorated and then improved. The authors suggest this implicates a hormonal aetiology.

#### Tinnitus

One study investigated tinnitus in pregnancy^7^ using a questionnaire and, by comparing 87 women with low-risk pregnancy with 47 non-pregnant women, found that the report of tinnitus is significantly more common in pregnancy: 25 per cent compared with 11 per cent. The authors theorised that the mechanism for this could be raised peri-lymph fluid pressure in pregnancy. A case report is described in which one patient’s tinnitus in pregnancy (with normal examination and imaging) became intolerable, necessitating caesarean section, after which all symptoms resolved.^19^

#### SSNHL

Many publications considered SSNHL in pregnancy, largely from the perspective of establishing safe treatment guidelines. Intra-venous Dextran, a plasma expander, is widely used in China and Taiwan, where patients are commonly admitted for treatment of SSNHL. Several case series show good effect from this treatment in pregnant women.^20^^,^^21^ The intention is to decrease blood viscosity and increase circulation to the cochlea, where microthrombi may have caused hypoxia. However, in their literature review, Xie and Wu acknowledged that there is a risk of pulmonary oedema with this intra-venous therapy.^6^

A British case report from 1998 reported good hearing improvement in pregnant women experiencing sudden sensorineural hearingloss with carbogen (5 per cent carbon dioxide, 95 per cent oxygen),^22^ but this has not been explored further in larger studies. Similarly, oral steroids were prescribed in two case reports,^23^^,^^24^ both with good audiological outcomes; however, concerns about fetal harm have prevented larger scale trials.

Fu *et al*.^25^ found intra-tympanic dexamethasone to be safe in a pilot study involving six pregnant women with SSNHL, as did Chen *et al*. in a single case report.^26^ A retrospective study of 30 patients with SSNHL in pregnancy, of whom half had been treated with intra-tympanic steroids in addition to Dextran, found that the intra-tympanic group showed significantly better hearing improvement despite similar demographics, with a mean PTA gain of 27.1 dB ± 16.4 compared with 15.7 dB ± 12.0 dB (*p* = 0.042).^27^ These results are supported by a similar retrospective study from 2020 involving 11 patients.^28^

In terms of the effect of pregnancy on outcomes of SSNHL, one prospective study enrolled 102 pregnant women with SSNHL and 102 non-pregnant patients with SSNHL as controls. They found that the hearing improvement after treatment was poorer in the pregnant group, but acknowledged that the two groups’ treatment regimens differed, with non-pregnant patients given a longer course of oral steroids and other additional therapies.^29^

Two smaller retrospective studies found no difference in hearing outcomes for pregnant and non-pregnant patients with SSNHL.^30^^,^^31^ One of these did, however, find that the fibrinogen level^32^ was significantly higher in their pregnant than non-pregnant SSNHL subjects.^30^

Several studies addressed the reason that SSNHL occurs in pregnancy. One case series (Hou 2011) described two patients with similar presentation, neither of whom were treated with steroids. One patient’s hearing loss recovered, while the other showed no improvement, and the authors propose two separate syndromes: a Meniere’s-like phenomenon caused by cochlear fluid variation and an ischaemic clinical picture resulting in permanent injury.

A published response to a case report made a link with studies that have shown SSNHL to occur in individuals with lower baseline blood pressure than controls^33^ and proposed that the pattern of variation of blood pressure during pregnancy – a fall followed by a rise – could create the conditions for SSNHL in some. The proposed aetiology is an abnormal vasomotor reaction to the episode of hypotension. A literature review published in the *Journal of Laryngology and Otology* in 2012 discussed possible aetiological theories: a direct effect of circulating sex hormones; the hypercoagulable state of pregnancy causing inner ear ischaemia; and water retention causing fluid retention and having a Meniere’s-like effect.^8^ Another review summarised findings of several of the above studies, noting again the clear benefit of intra-tympanic steroids.^34^

Two studies, one from Taiwan^35^ and one from South Korea^36^ used whole-population insurance data to analyse the incidence of SSNHL and concluded that it occurs *less* frequently in pregnancy than outside pregnancy, with no difference between incidence in the peri-partum and control groups. This suggests that, while SSNHL in pregnancy is of interest due to the nature of the presentation and the question of safe, effective treatment, studies discussing why this disease occurs in pregnancy may be subject to observer bias. Another study from South Korea used national records to compare women who developed SSNHL in pregnancy with those who were pregnant but did not develop SSNHL.^37^ Pre-pregnancy body mass index (BMI) was the only factor found to be associated with increased risk of SSNHL in pregnancy, with an adjusted odds ratio of 1.52 (95 per cent confidence interval 1.04-2.22). The patients with SSNHL were not at higher risk of pregnancy complications such as premature delivery and gestational hypertension. The authors suggest that the pro-inflammatory state of the metabolic syndrome, with insulin resistance and lipolysis, can contribute to a higher risk of thrombosis.

#### Auditory processing

Several studies assessed auditory processing in pregnancy, with some statistically significant findings as follows. One study that measured auditory brainstem responses (ABR) in eight pregnant women and 10 age-matched controls found an increase in the decibel threshold needed to elicit a response in pregnancy and an increased interpeak latency between wave I and wave V, indicating delayed neural conduction in pregnancy.^38^ A similar study from Iran found delayed interpeak latencies, as well as increased absolute latency for wave V.^39^ These authors reported that the effects were most marked when comparing pregnant women with controls in the early phase of the menstrual cycle, when oestrogen and progesterone levels are lowest, and suggested sensitivity of the auditory brainstem pathway to sex hormones, possibly via GABA neurotransmission in the brainstem. Another study also tested ABR in women at different phases in the menstrual cycle and found wave III latency to be delayed when oestrogen level was highest: the authors propose that oestrogen reduces blood flow in the brainstem.^40^

Where auditory cortex responses were tested, Begum and Reza found some waves to be reduced in amplitude with no significant differences in latency^41^ and, in auditory attention and executive function tests, found pregnant subjects to score more highly than non-pregnant controls. Slow vertex responses (SVR) were also slowed in pregnancy^42^ and the authors of this study remark that effects on central processing are unlikely to be mediated by electrolyte variation and fluid shifts in pregnancy.

#### Vestibular function

There is limited literature on the effect of pregnancy on vestibular function. A questionnaire completed by 82 pregnant women with no previous ear complaints found that 95 per cent of those in the first trimester reported nausea, with 48 per cent still affected in the third trimester.^43^ A total of 12 per cent reported vertigo, while 13 per cent reported instability and 11 per cent an unbalanced gait. A retrospective review of 68 pregnant patients presenting to ENT in Taiwan found that half were diagnosed with vestibular migraine, while others had new diagnoses such as Meniere’s disease and labyrinthitis. Of 32 vestibular evoked myogenic potentials (VEMPs) performed in this same study population (which assess utricle and saccule function through the vestibulo-ocular and vestibulo-collic reflexes), 13 were abnormal.^44^ A study that compared VEMPs in 17 pregnant and 17 non-pregnant women found that peak to peak amplitude was significantly reduced in the first trimester when compared with non-pregnant women.^45^ This raises the possibility that vestibular dysfunction may contribute to nausea and vomiting in early pregnancy. Another, uncontrolled study recorded the video head impulse test (VHIT) on pregnant women to assess lateral semicircular canal function via the vestibulo-ocular reflex. They found that the gain – the speed at which the eyes turn in response to a head movement – increased over the course of pregnancy, again suggesting minor vestibular dysfunction in the first trimester.^46^

#### Vestibular disorders

There is minimal examination of vestibular disorders in pregnancy in the literature. The challenge of interpreting case studies is well illustrated by comparing a report of a patient with Meniere’s disease whose vertigo attacks became more frequent during two separate pregnancies^47^ with another report describing Meniere’s-associated hearing loss that improved in two consecutive pregnancies and returned to baseline after delivery.^48^ Another case study describes acute vestibular neuritis in pregnancy without hearing loss, which was treated and resolved with no discernible link to the pregnancy.^49^

A literature review of vertigo in pregnancy that incorporated articles in English and Spanish found evidence that 57 percent of Meniere’s disease patients and 50 percent of patients with vestibular migraine experience flare-ups in the third trimester.^50^ The authors also comment that vestibular symptoms in pregnancy are likely to be attributed to pregnancy and not investigated. A descriptive chapter on vestibular migraine reports anecdotally that menstrual migraine sufferers often experience improvement during pregnancy, which could relate to a cessation of cyclical hormonal variation.^51^ A literature review from the United States also notes that vestibular disorder symptoms can fluctuate according to the menstrual cycle and proposes a vestibular cause for nausea and vomiting of pregnancy.^52^ The review suggests that this pathology may share an aetiology with multiple sclerosis, which often involves vestibular dysfunction in its early stages.

#### Cerebellopontine angle lesions

Lesions of the cerebellopontine angle (CPA) in pregnancy were a common theme of case studies due to challenging management decisions. Several of these reports describe a history of existing symptoms, such as hearing loss, with a sudden progression to hydrocephalus in pregnancy.^53^ Four patients with vestibular schwannoma presented with symptoms of hydrocephalus necessitating ventriculoperitoneal shunting prior to emergency Caesarean section ^54^^–^^56^ or emergency Caesarean section alone.^57^ Meningioma has also presented in a similar way.^58^ One of these presentations was initially misdiagnosed as pregnancy-related headache and vomiting, a reminder of the need for vigilance in clinical assessment.^56^

The authors of one of these case reports suggest two theories for accelerated tumour growth during pregnancy: increased blood flow to the lesion and tumour growth mediated by circulating sex hormones.^54^ A case series traced progression of symptoms in eight women who had vestibular schwannoma in pregnancy and reported that seven had a worsening of symptoms in late pregnancy.^59^ However, this pattern did not recur in subsequent pregnancies for the same patients, and a histological study of schwannoma tumour tissue in pregnant women, non-pregnant women and men found no statistically significant association between oestrogen or progesterone receptor quantity and tumour size, or DNA activity.^60^ This study comments that, as in the example of SSNHL, case reports that describe a phenomenon occurring during pregnancy can, without evidence, imply causation.

### Pregnancy syndromes and the inner ear

Eleven articles investigated the function of the inner ear in pre-eclampsia and other syndromes of pregnancy, all of which were prospective observational studies. The majority concerned hearing, while two assessed vestibular function.

#### Pre-eclampsia

Three studies performed PTA in pregnant women with and without pre-eclampsia and found that thresholds were worse in pre-eclampsia to a statistically significant degree in multiple frequencies.^61^^–^^63^ One of these studies repeated the assessment after delivery and found the changes to persist (Baylan 2010). The authors attributed these changes to the endothelial dysfunction that occurs when antiangiogenic factors proliferate in pre-eclampsia. This endothelial dysfunction leads to proteinuria and hypertension and may also cause microangiopathy and ischaemia in the inner ear.^61^ These changes can persist for years after delivery and cause higher rates of cardiovascular disease in patients with a history of pre-eclampsia.

Another study found transient evoked otoacoustic emissions (TEOAE) abnormalities in five out of 37 pre-eclampsia patients and zero out of 38 pregnant controls, but did not report the stage of gestation when the assessments were made.^64^ A study from Indonesia reported no difference in otoacoustic emissions (OAE) or tympanometry findings between pre-eclamptic and normotensive women two days after delivery.^65^ Altuntaş *et al*.^66^ tested TEOAE and PTA one week post-partum and found no difference between a group of 107 women who had had an uncomplicated pregnancy and a group of 97 women with various hypertensive pregnancy syndromes (pre-eclampsia, eclampsia, HELLP (haemolysis, elevated liver enzymes, low platelet count) syndrome and gestational hypertension).

A case series from Canada described a patient who developed tinnitus in the third trimester in the context of pre-eclampsia.^67^ The authors linked this case with two reports of Bell’s palsy in the third trimester in pre-eclampsia and suggested that neural oedema may be the cause.

#### Hyperemesis gravidarum

Two studies investigated vestibular function in patients with hyperemesis gravidarum. When testing high-frequency semicircular canal activity using the video head impulse test (VHIT), Tulmaç *et al*.^68^ did not find any significant difference in any canal between a group of 36 women with hyperemesis and 34 without, all in the first trimester. However, at lower frequencies of vestibular activity, a study employing the vestibular autorotation test found significant differences between hyperemesis patients, those with nausea and vomiting of pregnancy (NVP) and pregnant women who were not nauseous.^69^ Those with hyperemesis had higher horizontal gain in the vestibulo-ocular reflex (VOR), and this remained high in the peri-partum review, leading to a hypothesis that women with subclinical VOR abnormalities are susceptible to hyperemesis and NVP. The authors report that women who suffer with NVP are more likely to give a history of migraine and motion sickness which may also suggest predisposing vestibular pathology, raising the possibility of pre-pregnancy testing to determine those at risk.

Hyperemesis gravidarum was not found to affect PTA thresholds when compared with first-trimester pregnant controls.^70^

#### Gestational diabetes mellitus

A PTA study of gestational diabetes found significantly worse thresholds in the higher frequencies (8 to 14 kHz) when compared with pregnant controls, but no difference in the average PTA threshold.^71^ The authors attribute this finding to microangiopathy caused by hyperglycaemia, supported by the lack of reversibility they found when testing again at four weeks post-partum.

Another study assessed auditory processing by testing auditory evoked responses (AER) in twenty women with gestational diabetes and twenty pregnant controls, matched for age and gestational age.^72^ The absolute latencies of peaks I to V of the auditory brainstem response (ABR) were increased and the wave V amplitude was decreased in gestational diabetes. There was no significant difference in the mid-latency responses (MLR), which measure thalamocortical projections, but the SVR, which measure the auditory cortex response along with temporoparietal association areas, again showed significantly prolonged latencies of all components. This is evidence of slowed neural conduction time and central neuropathy in gestational diabetes, which could be due to microangiopathy or impairment of the electroconductive properties of myelin.

## Discussion

There are multiple limitations when gathering data that concerns pregnancy. Randomised controlled trials are generally not considered ethical; therefore, the best evidence comes from controlled observational studies. A case report may be published when a memorable patient presents with an auditory complaint in pregnancy, even though the pregnancy may not be responsible for their symptoms. It is also likely that publication bias has prevented the publication of negative findings in pregnancy and pregnancy syndromes.

However, despite these limitations, a wide-ranging review of the available literature has found evidence of statistically significant changes in hearing in pregnancy, pre-eclampsia and gestational diabetes. Other literature reviews, while commenting on the heterogeneity of studies and poor quality of much evidence, have nonetheless concluded that small, transient losses of hearing occur in pregnancy.^73^^,^^74^

Pregnancy may uncover pre-existing vestibular pathology, leading to nausea and vomiting or even hyperemesis gravidarum, and Meniere’s disease and vestibular migraine may be exacerbated by pregnancy.

- The inner ear is a complex sensory organ with finely balanced physiology
- Pregnancy involves multiple significant reversible alterations in physiological state, offering a potential model for measuring the impact of these alterations on hearing and balance function
- The literature demonstrates consistent evidence of subclinical hearing loss in pregnancy, which resolves following childbirth
- There is evidence that vestibular dysfunction in pregnancy may contribute to pregnancy-associated nausea
- Sudden sensorineural hearing loss (SSNHL) does not occur more frequently in pregnancy

### Aetiological theories

During pregnancy, oestrogen and progesterone are produced initially by the corpus luteum and later by the placenta. As the levels of these sex hormones rise, they exert effects on virtually every body system. Changes in the cardiovascular, coagulation and immune systems are most pertinent to this review. Circulating blood volume increases by 30 to 40 per cent during pregnancy, and cardiac output increases by between 30 and 50 per cent. There is a drop in total peripheral resistance by approximately 20 per cent. As these changes occur, blood pressure drops and pulse pressure widens in the first trimester, and then returns to baseline in the second half of a healthy pregnancy. There is a degree of haemodilution in pregnancy as plasma volume increases at a higher rate than red blood cell count. Serum sodium and serum osmolarity are slightly reduced, but the concentration of other electrolytes is not affected by pregnancy. Multiple studies included in this review suggested that increased circulating volume could affect the fluid balance in the inner ear, and the electrolyte balance between endolymph and peri-lymph, with outcomes similar to that of endolymphatic hydrops in Meniere’s disease. However, the theory that sodium retention could explain these changes is less convincing given that plasma sodium concentration does not increase but decreases in pregnancy. No animal study has yet investigated these theories by measuring peri-lymph and endolymph composition in pregnancy.

Pregnancy is a hypercoagulable state, with a four- to five-fold increase in the incidence of thrombosis in pregnancy compared with the non-pregnant state. This is believed to be an evolutional adaptation reducing the risk of catastrophic haemorrhage during delivery and peri-partum. The mechanism of hypercoagulability is attributed to an increase in plasma levels of clotting factors, increased platelet reactivity and stasis due to compression of the great veins by the gravid uterus.

The possibility of microthrombi or emboli in the labyrinthine artery or its branches is mentioned by several studies in this review as a mechanism through which hearing loss and vestibular pathology may occur in pregnancy.

Multiple changes occur in the maternal immune system during pregnancy resulting in increased tolerance towards foreign antigens, which allows the fetus and placenta to avoid rejection. This immune tolerance is believed to explain the increase in susceptibility to contracting infection and the increased severity of certain infections during pregnancy. Immune tolerance in pregnancy may lead to viral reactivation affecting the vestibular and cochlear nerve and could cause sudden sensorineural hearing loss or acute vestibular neuritis.

Another theory is that pregnancy hormones exert effects directly on the structures of the inner ear, altering sensitivity to auditory stimuli, as well as to head position and movement. This could account for the reversibility of changes to inner ear function reported by some studies. A histological study from 2001 found oestrogen alpha receptors in the spiral ganglion and oestrogen beta receptors in the stria vascularis,^75^ giving some credibility to this hypothesis.

Future research that would advance our understanding of the ear in pregnancy would include a pre-registered large-population multicentre observational study in which pure tone audiogram, auditory brainstem responses and vestibular function (with video nystagmography and video head impulse test) are recorded and traced through pregnancy and post-partum, with matched non-pregnant controls. This would elucidate the nature of pregnancy-induced hearing loss and eradicate the publication bias that may be distorting the conclusions drawn by this review. A similar dataset of patients with hypertensive pregnancy disorders and gestational diabetes would be equally valuable for understanding the effect of these syndromes on the inner ear.

## Conclusion

The literature clearly demonstrates evidence of subclinical reversible hearing loss during healthy pregnancy, and currently the best explanation for this is a change in electrolyte balance in the endolymph and peri-lymph system of the cochlea. Auditory processing also appears to be affected by pregnancy, with changes to latency and amplitude of responses: the proposed theory is that pregnancy hormones may alter neuronal conduction or intra-cranial blood flow. There is some evidence that vestibular function alters during pregnancy, particularly in patients suffering from hyperemesis gravidarum. There is no evidence that SSNHL occurs more frequently in pregnancy; its emergence in the literature focusses largely on safe management.

The literature discussing the effect of pregnancy syndromes on the inner ear is limited, but the studies that exist suggest this could be a fruitful area for further study. Pre-eclampsia and gestational diabetes appear to have a greater effect on hearing than pregnancy alone, and this effect may be permanent.

## Data Availability

All systematic review data are included within the manuscript (some may be more appropriate as supplementary files than inclusion in the final manuscript).
